# Educational Formats and Content Domains of Interprofessional Education for Licensed Rehabilitation Professionals: Scoping Review

**DOI:** 10.2196/76189

**Published:** 2026-03-04

**Authors:** Kohei Ikeda, Takao Kaneko, Someka Hijikuro, Natsuki Inoue, Takuto Nakamura, Taisei Takeda, Junya Uchida, Hirofumi Nagayama

**Affiliations:** 1 Division of Occupational Therapy Program, School of Rehabilitation, Faculty of Health and Social Service, Kanagawa University of Human Services, 1-10-1 Heiseicho, Yokosuka, Kanagawa, Japan, 81 468282803; 2 Department of Rehabilitation, Yamagata Prefectural Central Hospital, Yamagata, Japan; 3 Department of Rehabilitation Therapy, Saiseikai Higashikanagawa Rehabilitation Hospital, Yokohama, Japan; 4 Dalla Lana School of Public School, University of Toronto, Toronto, ON, Canada

**Keywords:** educational content domains, interprofessional education, licensed rehabilitation professionals, educational formats, scoping review

## Abstract

**Background:**

Interprofessional education (IPE) is a key strategy for enhancing collaboration and patient safety. While evidence for student populations is abundant, studies focusing on licensed physical therapists (PTs), occupational therapists (OTs), and speech-language pathologists (SLPs) remain limited. In contemporary rehabilitation practice, continuous IPE is increasingly important to address professional burnout and the growing complexity of patient needs.

**Objective:**

This scoping review aimed to systematically map and synthesize the educational formats, content domains, and reported outcomes of IPE programs specifically targeting licensed PTs, OTs, and SLPs.

**Methods:**

Following Joanna Briggs Institute and PRISMA-ScR (Preferred Reporting Items for Systematic Reviews and Meta-Analyses extension for Scoping Reviews) guidelines, we searched the PubMed, Web of Science, Cumulative Index to Nursing and Allied Health Literature, and Educational Resources Information Center databases through December 31, 2025. The eligibility criteria were based on the population, concept, and context framework, including peer-reviewed, English-language studies of licensed PTs, OTs, and SLPs (population) participating in structured IPE interventions (concept) within clinical or community settings (context). Studies focusing solely on students or prelicensure trainees were excluded. Following the screening of 3234 records by independent pairs of reviewers, 9 studies were ultimately selected for inclusion. Methodological quality was appraised using Joanna Briggs Institute critical appraisal checklists and the Mixed Methods Appraisal Tool. Data were synthesized using an evidence gap map to visualize research density across domains relative to established competency frameworks.

**Results:**

A total of 9 studies from Australia, the United States, Canada, and the Philippines were included, with sample sizes ranging from 8 to 197. Most used single-group pre-post or mixed methods designs; notably, no randomized controlled trials were identified. Methodological quality was generally high, though limited by the lack of control groups. Systematic mapping identified 7 educational formats, with lectures and discussions being the most dominant across all competency domains. Primary content domains included communication and role clarification. Specific successful interventions included pharmacist-led medication safety workshops and the Kawa model for team building. While participants reported immediate improvements in role understanding and collaborative confidence, simulation-based training showed inconsistent effects on long-term clinical behavior. A substantial evidence gap was identified in experiential learning approaches targeting collaborative leadership.

**Conclusions:**

This scoping review adds a novel perspective by focusing exclusively on licensed rehabilitation professionals (PTs, OTs, and SLPs), highlighting learning needs distinct from those of prelicensure students. It brings to the field a clearer understanding of a potential “leadership gap” and the current overreliance on didactic methods for experienced clinicians. Real-world implications suggest the need for health care institutions to transition toward systematic, practice-integrated IPE models that incorporate objective behavioral assessments. By addressing identified gaps in collaborative leadership and team functioning through longitudinal programs, health care institutions may contribute to more resilient team cultures, ultimately improving patient safety and the quality of rehabilitation care.

## Introduction

Patient safety remains a global priority in health care systems worldwide. The World Health Organization estimates that approximately 1 in 10 patients receiving medical care experiences an adverse event, more than half of which are considered preventable [1]. Communication failures among health care professionals, as well as between professionals and patients or families, are consistently identified as a major contributing factor, accounting for nearly 70% of adverse events [1,2]. Consequently, improving interprofessional communication and collaboration has been recognized as a key strategy for enhancing patient safety and quality of care [2-4].

Interprofessional education (IPE) has been promoted as an effective educational approach to address these challenges. The World Health Organization [1] defines IPE as occasions when “two or more professions learn about, from, and with each other to enable effective collaboration and improve health outcomes.” A growing body of literature demonstrates that IPE can improve communication skills, mutual understanding of professional roles, collaborative attitudes, and teamwork among health care students and trainees [5-8]. IPE also deepens understanding of the expertise and responsibilities of other health professionals [5,6,9]. In addition, previous studies have reported positive downstream effects of IPE on clinical practice, including improved teamwork in intensive care settings, reduced fall risk in patients, and more efficient patient transfers [7].

Within rehabilitation settings, collaboration among physical therapists (PTs), occupational therapists (OTs), and speech-language pathologists (SLPs) is particularly critical, as patient care often requires coordinated, goal-oriented interventions across disciplines. Interprofessional collaboration (IPC) in rehabilitation has been associated with improved functional outcomes, enhanced patient safety, and greater continuity of care [10-12]. Importantly, IPE for licensed rehabilitation professionals differs from student-based IPE, in that it emphasizes experiential learning grounded in clinical practice, professional identity, and role negotiation within real-world care environments [13]. Recent literature further emphasizes that while prelicensure IPE focuses on fundamental attitudinal changes—such as fostering mutual respect and role awareness among students—postlicensure IPE for practicing clinicians is a vital tool for addressing contemporary challenges, such as the increase in clinician burnout and the rising complexity of geriatric and neurological rehabilitation [14,15]. Furthermore, updated international frameworks, such as the 2023 interprofessional education collaborative core competencies, now explicitly highlight the need for practice-integrated learning to foster a resilient team culture and improve staff retention in high-pressure clinical environments [16,17]. Despite its recognized importance, evidence suggests that structured IPC remains insufficiently implemented in rehabilitation practice. A qualitative study conducted in an Irish rehabilitation hospital reported that many teams identifying as interdisciplinary lacked regular opportunities for shared goal-setting and reflective dialog [18]. Similarly, a Norwegian national survey revealed substantial variability in team communication and coordination, indicating that consistent interprofessional practices are not yet well established across rehabilitation settings [19]. The need for a more robust evidence base is underscored by the current global shortage of rehabilitation professionals, which demands more efficient, collaborative delivery models to maintain care quality. These findings highlight the persistent gap between the conceptual ideal of interprofessional teamwork and its practical realization in clinical rehabilitation.

Although interest in IPE has increased over the past decade, most existing research has focused on prelicensure health care students. Evidence regarding IPE for licensed rehabilitation professionals remains limited and fragmented. Previous reviews by Spaulding et al [8] and Shuyi et al [7] identified only a small number of studies targeting practicing clinicians and did not comprehensively examine how such programs are structured, what educational content they include, or how outcomes are evaluated. Furthermore, few reviews have used integrative visualization tools, such as evidence gap maps, to pinpoint specific intersections between educational formats and competency domains in this specific professional cohort. Given the diversity of professional roles, clinical settings, and educational approaches within rehabilitation, a broad mapping of existing evidence is warranted.

Therefore, this scoping review aimed to systematically map and synthesize the existing literature on IPE for licensed PTs, OTs, and SLPs. Specifically, this review sought to address the following research questions:

1. What are the structures (eg, target group, format, frequency, or duration) of IPE programs for licensed PTs, OTs, and SLPs?
2. What educational content (eg, themes, skills, or learning objectives) is addressed in these programs?
3. How are these structures and contents evaluated, and what impacts have been reported?

By clarifying current practices and evidence gaps, this review seeks to inform the design of future IPE initiatives and contribute to strengthening IPC in rehabilitation settings.

## Methods

### Design

This scoping review was conducted in accordance with the methodological framework originally proposed by Arksey and O’Malley [20] and subsequently refined by Levac et al and the Joanna Briggs Institute (JBI) [21,22]. This approach is well-suited for mapping the extent, range, and nature of evidence across a broad topic area; identifying research gaps; and clarifying key concepts. Given the exploratory aim of this study—to examine the implementation and educational content of IPE for licensed PTs, OTs, and SLPs—a scoping review was deemed the most appropriate method.

The reporting of this review followed the PRISMA-ScR (Preferred Reporting Items for Systematic Reviews and Meta-Analyses extension for Scoping Reviews) guidelines [23,24] (Checklist 1; 2 ). In addition, the search strategy and reporting of the literature search process adhered to the PRISMA-S (Preferred Reporting Items for Systematic Reviews and Meta-Analyses Search Reporting Extension) guidelines to ensure transparency, reproducibility, and comprehensive documentation of all search components, including databases searched, search strategies, and limits applied [25] (Checklist 3).

**Figure 1.**
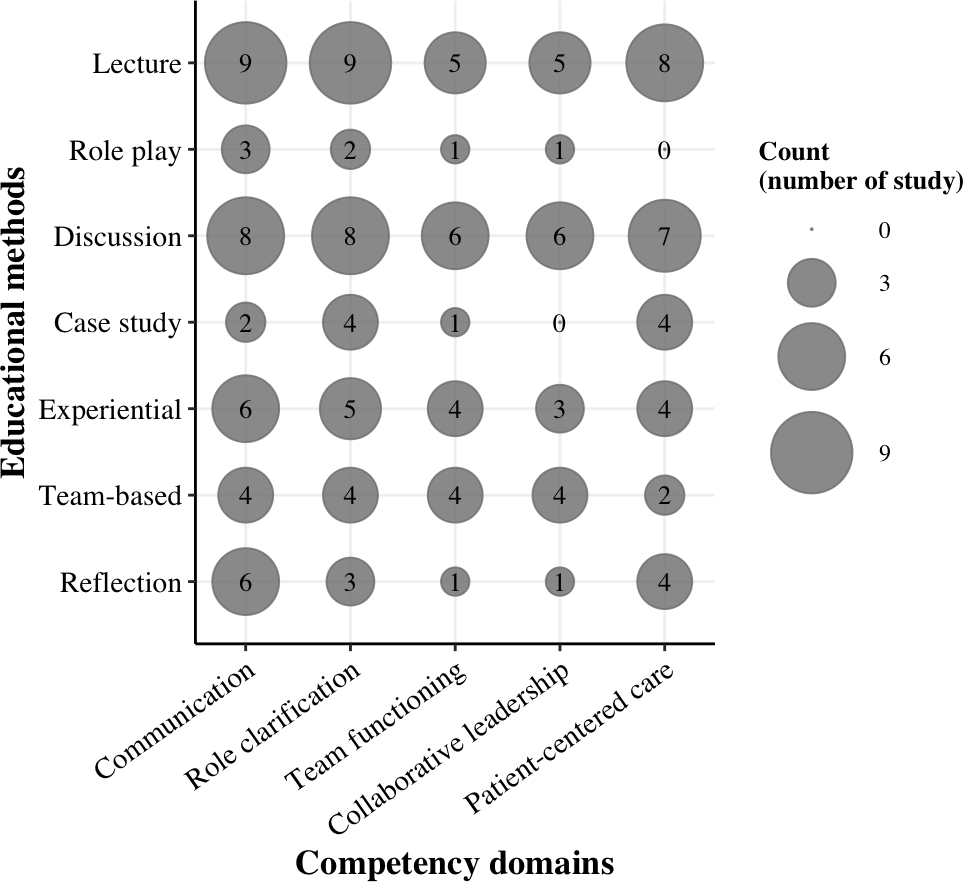
Evidence gap map of interprofessional education learning formats and competency domains.

The review protocol was prospectively registered on the Open Science Framework (DOI: 10.17605/OSF.IO/G5HQN). Furthermore, a preprint of this review has been made publicly available (DOI: 10.2196/preprints.76189) to enhance transparency, promote early dissemination, and allow for public scrutiny of the methodology. There were minor deviations from the original study protocol registered on Open Science Framework. Specifically, while the protocol initially planned to present findings primarily through descriptive tables, we expanded the visualization to include an evidence gap map (Figure 1) and thematic clustering of educational content (Table 1). These additions were made to provide a more intuitive and comprehensive overview of the research landscape and to better highlight identified gaps in the literature.

**Table 1.** Mapping of interprofessional education competency domains, outcome categories, and evaluation measures across included studies.

Author	Competency domains					Outcome categories				Measurement instruments				
	Communication	Role clarification	Team Functioning	Collaborative leadership	Patient-centered care	Knowledge	Attitudes	Confidence	Practice change	ATHCTS^a^	ISVS^b^	TSS^c^	IPC^d^	Other measures^e^
Eskola et al [26]	✓^f^	✓	✓	✓	✓		✓		✓					✓
Day et al [27]	✓	✓	✓	✓	✓		✓	✓	✓		✓			
Cohen et al [28]	✓	✓	✓	✓	✓		✓		✓	✓		✓		
Zhang et al [29]	✓	✓			✓	✓	✓	✓	✓	✓				
Ober & Lape [30]	✓	✓	✓	✓	✓	✓	✓	✓	✓	✓		✓		
Scuderi & Pain [31]	✓	✓			✓	✓		✓			✓			
Sy [32]	✓	✓	✓		✓		✓			✓			✓	
Phillips et al [33]	✓	✓	✓	✓	✓	✓	✓	✓	✓		✓			
Kenaszchuk et al [34]	✓	✓	✓	✓	✓	✓	✓							✓

a ATHCTS: Attitudes Towards Health Care Teams Scale.

b ISVS: Interprofessional Socialization and Valuing Scale.

c TSS: Team Skills Scale.

d IPC Scale: Interprofessional Collaboration Scale.

e Consultative and Relational Empathy, Interpersonal Reactivity Index, confidence-based marking, self-assessment tools used Likert scales to assess knowledge, confidence, attitudes, perceived practice changes, etc.

f Indicates that the domain or outcome was addressed in the study.

### Study Eligibility (Inclusion and Exclusion Criteria)

To ensure a systematic selection process, inclusion and exclusion criteria were structured according to the population, concept, and context framework [22]. The population, concept, and context framework is the recommended standard for scoping reviews to clearly define the boundaries of the inquiry beyond the traditional population, intervention, comparison, outcome format, which is often too restrictive for mapping broad educational interventions. This framework was selected to effectively capture the specific professional cohorts (population), the diverse pedagogical structures of IPE (concept), and the varied clinical environments of rehabilitation (context).

### Participants (Population)

This review focused on licensed rehabilitation professionals—specifically PTs, OTs, and SLPs—working in rehabilitation, health care, or community-based settings. These professionals play critical roles in improving patient function and quality of life, and their collaboration is vital for preventing adverse events such as falls and enhancing care quality [10-12]. PTs primarily address physical rehabilitation, OTs focus on daily living activities, and SLPs manage communication and swallowing functions. Their complementary expertise contributes to comprehensive patient care and supports the effectiveness of IPE initiatives [7,11]. Studies involving health care students, prelicensure trainees, or nonprofessional caregivers were excluded, as the objective of this review was to analyze IPE among qualified practitioners.

### Concept

Eligible studies were those investigating IPE programs involving at least 1 licensed PT, OT, or SLP. IPE was defined as a structured educational intervention delivered through various methods—such as lectures, workshops, simulations, or on-site training—aimed at improving IPC and communication. Studies were excluded if they lacked a clear description of the IPE content, implementation frequency, or reported outcomes.

### Context

Eligible studies were conducted in clinical practice environments, including rehabilitation facilities, hospitals, and community-based care settings. The context was restricted to real-world clinical or community practice environments; studies conducted exclusively in academic or simulation-only settings without relevance to licensed clinical practice were excluded.

### Study Design and Publication Type

Eligible study designs included intervention studies (eg, pre-post, quasi-experimental, and randomized controlled trials [RCTs]) and observational studies. Publications were original, peer-reviewed research articles written in English. Descriptive reports, theoretical papers, standalone qualitative studies, dissertations, conference proceedings, and letters were excluded.

### Rationale for Excluding Qualitative Studies

Although scoping reviews typically encompass a variety of study designs, this review prioritized studies allowing statistical comparison and synthesis of quantifiable outcomes. To maintain a consistent analytical framework focused on measurable effects of IPE, qualitative studies were excluded. Nonetheless, the complementary value of qualitative research is acknowledged for future synthesis.

### Search Strategy

A comprehensive literature search was conducted using four databases: PubMed, Web of Science, CINAHL (Cumulative Index to Nursing and Allied Health Literature), and Educational Resources Information Center —covering the period from the inception of each database up to December 31, 2025. These databases were selected due to their comprehensive coverage of research related to IPE and health care professionals, with a particular focus on rehabilitation, nursing, and educational fields. Due to institutional restrictions, access to the Scopus database, which covers a broad range of scientific literature, was unavailable during the review period. PubMed served as the interface to access Medical Literature Analysis and Retrieval System Online-indexed records; Medical Literature Analysis and Retrieval System Online was not searched separately.

The initial search strategy was developed in PubMed using both controlled vocabulary (Medical Subject Headings terms) and free-text keywords, including “interprofessional education,” “rehabilitation,” “physical therapy,” “occupational therapy,” and “speech-language pathology.” Boolean operators (AND, OR) were used to refine the results. This PubMed strategy was then adapted for each database to reflect differences in indexing systems and search syntax—for example, Medical Subject Headings terms were replaced with CINAHL, headings in CINAHL, and equivalent keywords were applied in Web of Science and Educational Resources Information Center using appropriate truncation symbols and Boolean operators. The complete search strings for all databases are provided in Multimedia Appendix 1 to ensure transparency and reproducibility. Reference lists of included articles were also manually screened to identify additional relevant studies. To ensure that the review reflects the most recent scholarly contributions in the field, the initial search was updated on January 3, 2026, by rerunning the exact search strings across all 4 databases. This update extended the search period to include all relevant records published through December 31, 2025.

In accordance with PRISMA-S guidelines, further methodological details are disclosed to ensure transparency. Contacting study authors for additional data was not performed as the published reports provided sufficient information, and no additional methods, such as searching clinical trial registries, were used given the review’s focus on peer-reviewed literature. While a formal external peer review of the search strategy was not conducted, the strategy was developed and validated through internal pilot testing and consensus among the research team.

### Study Selection

All retrieved records were imported into Rayyan software (Qatar Computing Research Institute), and duplicates were automatically removed. Title and abstract screening were conducted independently by pairs of reviewers (HN and JU, TN and TK, NI and TT, SH and KI). Each reviewer’s decisions were blinded to their respective partner within the pair to ensure unbiased screening. Any discrepancies were resolved through consultation with a third reviewer. The same independent and blinded process was applied during full-text screening. The selection process is illustrated in the PRISMA (Preferred Reporting Items for Systematic Reviews and Meta-Analyses) flow diagram.

### Data Extraction and Analysis

A structured data-extraction form was pilot-tested. A total of 2 or more reviewers independently extracted data regarding study design, setting, participant characteristics, IPE format, frequency, outcome measures, and key findings. Methodological quality was appraised using the appropriate JBI critical appraisal checklists [35] and the Mixed Methods Appraisal Tool [36] for descriptive purposes (MultimediaAppendices 2  3).

To enhance clarity, educational formats were categorized according to the Centre for the Advancement of Interprofessional Education framework [37], and content was classified using the framework proposed by Reeves et al [38]. An evidence gap map (Figure 1) was generated to visualize the density of research across these categories.

## Results

### Study Selection

**Figure 2.**
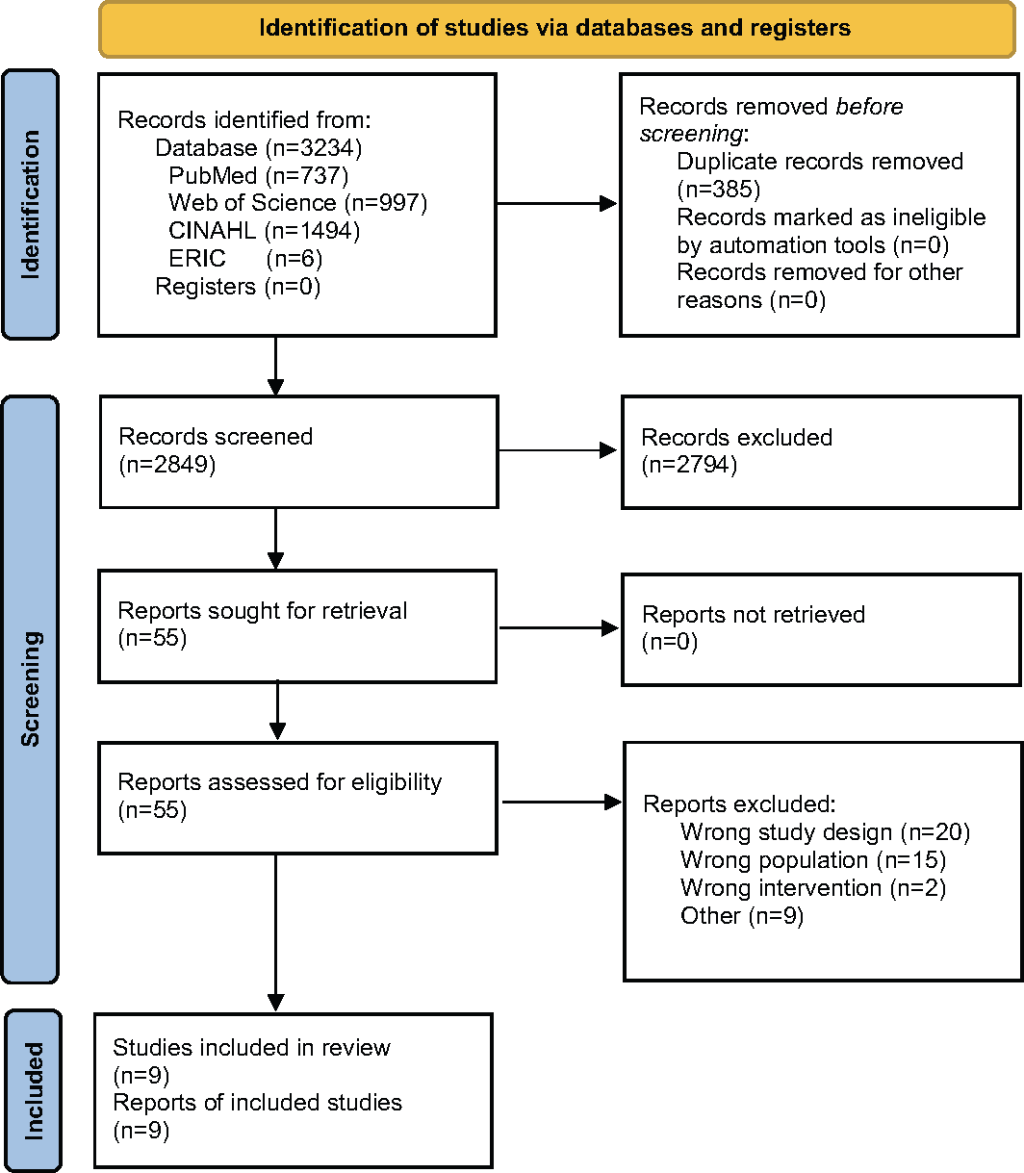
PRISMA (Preferred Reporting Items for Systematic Reviews and Meta-Analyses) flow diagram. ERIC: Educational Resources Information Center; CINAHL: Cumulative Index to Nursing and Allied Health Literature.

A total of 3234 articles were identified through database searches. After removing 385 duplicates, 2849 titles and abstracts were screened, resulting in the exclusion of 2794 articles. Full-text review of the remaining 55 articles led to the inclusion of 9 studies that met the eligibility criteria. One additional study was identified through the updated search conducted up to December 31, 2025. The list of excluded studies and their reasons for exclusion is provided in Multimedia Appendix 4. The study selection process is summarized in the PRISMA flow diagram (Figure 2).

### Study Characteristics

Of the 9 included studies, 1 used a cross-sectional design [32], 5 used single-group pre-post comparisons [28,29,31,33,34], and 3 adopted mixed methods combining pre-post and qualitative approaches [26,27,30] (Table 2). Notably, 1 pre-post study followed a quasi-experimental design [29]. Despite the rigorous selection process, no RCTs were identified.

**Table 2.** Characteristics of included studies.

Author, year, country	Study design	Setting	Population	Professions	Key findings
Eskola et al, 2024 [26], United States	Mixed methods pre-post	Veterans affairs hospital and clinics	71 clinicians	Nurses, providers (MD^a^, NP^b^, PA^c^, etc), SWs^d^, OTs^e^, and pharmacists	Significant improvements in listening, understanding patient concerns, and showing compassion (*P*<.05). Increased perceived value of peer observation and reflection. Improved ability to work within interprofessional teams.
Day et al, 2022 [27], Australia	Mixed methods process evaluation	9 organizations across Adelaide and Sydney (aged care, disability, and navigation services)	9 professionals (3 aged care, 3 disability, and 3 navigation)	Health services professionals, and OTs	High feasibility and completion; ISVS^f^-21 scores improved; NoMAD^g^ showed increased confidence but reduced perceived integration; qualitative data revealed enhanced understanding, motivation, and team-based implementation; sustainability supported by structured planning and peer learning.
Cohen et al, 2021 [28], United States	Quasi-experimental pre-post	5 regions in the United States (New York City, Phoenix, Chicago, North Carolina, and Washington DC)	236 practitioners	MD, RN^h^, NP, PA, OT, PT^i^, SLP^j^, MT^k^, and SW	Significant improvement in TSS^l^ scores overall (median 3.76 [IQR 3.4-4.1] to 4.35 [IQR 3.9-4.7]; *P*<.001); all professions improved except the PA group (n=3); 58% rated themselves “very likely” to change practice, and this group showed greater TSS gains (*P*=.03); TSS change correlated with ATHCTS^m^ (*ρ*=0.42), confidence with PD^n^ (*ρ*=0.18-0.21), team knowledge (*ρ*=0.23-0.29), and new information gained (*ρ*=0.26-0.31).
Zhang et al 2021 [29], Australia	Single-group pre-post	Pediatric hospital and health service in New South Wales	30 interpreters or translators	Interpreters and translators (various languages and cultural backgrounds)	Significant improvement in knowledge and confidence (pre→post); practice improved post but declined at the 2-month follow-up (2MPost); attitudes remained high throughout; less experienced ITs showed greater knowledge gains.
Zhang et al, 2021 [29], Australia	Quasi-experimental	Pediatric hospital and health service	49 SPs^o^ (27 training, 22 control)	SLPs	Training group showed significant improvements in knowledge, confidence, and practice (pre→post, pre→2MPost); practice declined at 2MPost; attitudes mostly unchanged except A1; training rated highly useful and informative.
Zhang et al, 2021 [29], Australia	Quasi-experimental	Pediatric hospital and health service	24 clinicians (16 training and 8 control)	Allied health (OT, PT, psychology, etc) and nursing	Training group showed significant improvements in Knowledge, confidence, and practice (pre→post, pre→2MPost); practice declined at 2MPost; attitudes unchanged; training rated useful, but application to practice was modest.
Ober & Lape, 2019 [30], United States	Mixed methods pre-post	1 suburban acute care hospital in Pennsylvania	8 rehabilitation professionals	PTs, OTs, and SLPs	Quantitative: All 7 items improved; familiarity with the Kawa model increased most (+3.13); team collaboration scores improved to mean 4.75 (SD not reported). Qualitative: Postsurvey responses showed deeper recognition of individual differences, expanded definitions of professionalism, and more creative collaboration strategies. The Kawa model facilitated shared language and mutual understanding.
Scuderi & Pain, 2019 [31], Australia	Observational (pre-post)	Acute care hospital	33 professionals (28 junior and 5 senior)	PTs, dieticians, SLPs, and OTs	Significant improvement in knowledge and confidence on 5/7 items; 93% satisfaction; CBM^p^ effective in promoting safe decision-making.
Sy, 2017 [32], Philippines	Cross-sectional survey	National (online survey of licensed professionals in the Philippines)	189 total (OT: 100, PT: 56, and SLP: 33)	OTs, PTs, and SLPs	Prior IPE^q^ experience associated with more positive IPC^r^ attitudes; lecture and CBR^s^ case studies linked to higher AI^t^ scores.
Phillips et al, 2016 [33], Australia	Quasi-experimental (pre-post + 3 mo follow-up)	45 workshops across 7 states/territories via Medicare locals	837 participants (645 enrolled in evaluation)	GPs^u^, nurses, mental health professionals, OT, PT, pharmacists, podiatrists, and social workers	Significant improvements in knowledge and confidence; increased use of psychological strategies (especially GPs); 77% of planned practice changes achieved; increased network ties to psychologists, psychiatrists, VVCS^v^; attitudes toward interprofessional working improved.
Kenaszchuk et al, 2011 [34], Canada	Single-group pre-post (uncontrolled intervention)	Community hospital near Toronto	154 health professionals (physicians, nurses, and allied health)	Physicians, nurses, allied health (OT, PT, SLP, pharmacists, social workers, and dietitians)	Simulation training led to attitude improvements among those with lower initial IPC scores; shared leadership attitudes improved over time; reliability of measures varied by profession.

a MD: doctor of medicine.

b NP: nurse practitioner.

c PA: physician assistant.

d SW: social worker.

e OT: occupational therapist.

f ISVS: Interprofessional Socialization and Valuing Scale.

g NoMAD: Normalization Measure Development questionnaire.

h RN: registered nurse.

i PT: physical therapist.

j SLP: speech-language pathologist.

k MT: medical technologist.

l TSS: Team Skills Scale.

m ATHCTS: Attitudes Towards Health Care Teams Scale.

n PD: Parkinson disease.

o SP: standardized patient.

p CBM: confidence-based marking.

q IPE: interprofessional education.

r IPC: interprofessional collaboration.

s CBR: community-based rehabilitation.

t AI: artificial intelligence.

u GP: general practitioner.

v VVCS: Virtual Village Case Study.

Participants included a wide range of health professionals, such as OTs, PTs, SLPs, physicians, nurses, pharmacists, social workers, psychologists, dietitians, mental health specialists, podiatrists, prosthetists, and interpreters. Sample sizes ranged from 8 to 197. Training facilitators included clinicians and academic educators with formal teaching experience [28,30,31]. Recruitment strategies varied globally, with significant representation from Australia [27,29,33], the United States [26,30], Canada [34], and the Philippines [32].

### Implementation and Content

The educational formats and content domains of the IPE programs are summarized in Table 3 and visualized in our evidence gap map (Figure 1). Systematic mapping identified 7 educational formats. Didactic methods, specifically lectures (n=36 mentions across studies) and discussions (n=35), were the most dominant and were consistently applied across all competency domains. In contrast, experiential formats such as role play (n=7) and case studies (n=11) were notably less frequent.

**Table 3.** Characteristics of IPE^a^ formats.

Author	IPE format	Frequency	Trainer background
Eskola et al [26]	6.5-hour workshop (lecture, actor-based simulation, and applied improvisation)	Single-day session	Palliative care physicians and an experienced medical educator
Day et al [27]	6-module online education + webinars + mentoring + team-based implementation planning	Weekly sessions over 5 weeks	Delivered by university faculty with simulation teaching experience; professions included OT^b^, PT^c^, and nursing
Cohen et al [28]	ATTP-IPE^d^ training program (single session)	Once	Delivered by interprofessional faculty team; included MDs^e^, RNs^f^, and educators with IPE teaching background
Zhang et al [29]	120-minute face-to-face training (presentation, handouts, role play, and discussion)	Not applicable (retrospective survey)	Delivered by bilingual clinical educators with teaching experience; professions not specified
Ober & Lape [30]	5-week team-building intervention using Kawa model in individual and group sessions	One-time training per participant	Delivered by OT faculty with expertise in Kawa model and team facilitation
Scuderi & Pain [31]	2-hour pharmacist-led workshop with didactic + case study + CBM^g^-based quiz	Single session per participant	Delivered by clinical pharmacists with teaching experience
Sy [32]	Retrospective self-report of prior IPE experience; classified into 8 TL^h^ strategies (eg, case discussion, small group discussion, and lecture)	1 session per participant (12 workshops total)	Not applicable; no training delivered in study
Phillips et al [33]	6-hour or 2×3-hour workshop with didactic content, facilitator guide, and commitment-to-change	6-month engagement (mean 8.3 hours education)	Delivered by trained facilitators including GPs^i^, nurses, and mental health professionals; all had prior IPE facilitation experience
Kenaszchuk et al [34]	1-day interprofessional simulation workshop with didactic + experiential learning + debrief	Single session	Delivered by trained facilitators from participating professions; simulation experts with IPC^j^ background

a IPE: interprofessional education.

b OT: occupational therapist.

c PT: physical therapist.

d ATTP-IPE: allied team training for Parkinson interprofessional education.

e MD: doctor of medicine.

f RN: registered nurse.

g CBM: confidence-based marking.

h TL: teaching-learning.

i GP: general practitioner.

j IPC: interprofessional collaboration.

Regarding educational content, communication (n=38) and role clarification (n=35) were the most frequently addressed competencies. The evidence gap map (Figure 1) highlights a high density of research focusing on these foundational competencies via traditional didactic methods. Conversely, collaborative leadership (n=20) was the least addressed domain, representing a significant gap in the current IPE landscape, particularly regarding its implementation through experiential learning.

### Outcome Measures

Outcome evaluation focused primarily on short-term educational effects, such as changes in knowledge, attitudes, and confidence. A variety of validated instruments were used, including the Attitudes Toward Health Care Teams Scale [39], the Interprofessional Socialization and Valuing Scale [40], and the Team Skills Scale [41]. Several studies supplemented these with study-specific questionnaires or confidence-based marking to assess decision-making safety [29,33]. Long-term evaluations of behavioral changes in clinical practice or patient-level outcomes were rarely reported across the included studies.

### Main Findings

Overall, participants reported positive attitudes toward IPE. Lecture and case study formats were particularly effective in enhancing understanding of professional roles and medication management [30,31]. The Allied Team Training for Parkinson program demonstrated significant gains in perceived team skills and interprofessional understanding [28].

Temporal analysis showed that structured workshops yielded immediate gains in knowledge and confidence [29,33]. However, some inconsistent effects were noted; for instance, simulation-based training did not result in long-term attitudinal improvements [34]. Furthermore, community-based strategies occasionally faced challenges such as group tensions and mismatched professional expectations [27]. These findings underscore that while foundational knowledge is successfully transmitted through current IPE models, sustained behavioral change remains a complex challenge for licensed professionals.

## Discussion

This scoping review synthesized the implementation formats, content domains, and reported outcomes of IPE initiatives targeting licensed PTs, OTs, and SLPs, addressing our primary research questions regarding how these programs are structured and evaluated. We identified a clear predominance of didactic formats, such as lectures and discussions, which were most frequently applied to foundational competency domains, including communication and role clarification (Figure 1). While these initiatives generally improved participants’ understanding of professional roles and collaborative skills, the evidence suggests a notable scarcity of experiential or simulation-based approaches, particularly in complex domains such as collaborative leadership. These results suggest that although the “what” and “how” of IPE are well-documented for foundational skills, more advanced competencies remain insufficiently addressed, suggesting that the field has yet to fully embrace pedagogical methods that challenge the entrenched professional silos of established practitioners.

The findings of this scoping review extend the existing IPE literature by clarifying how educational formats and content domains are distributed within programs targeting licensed rehabilitation professionals. Previous reviews of IPE have predominantly focused on prelicensure learners and consistently reported positive effects of didactic teaching, simulation, and structured reflection on attitudes toward teamwork and role understanding [5-8]. In contrast, our review demonstrates that, although similar didactic formats dominate postlicensure IPE for practicing PTs, OTs, and SLPs, their reported effects are largely confined to short-term gains in knowledge, confidence, and role clarification. This divergence suggests that educational strategies effective for students may not directly translate to experienced clinicians, whose learning needs are shaped by established professional identities, ingrained clinical routines, and organizational constraints [42,43]. By mapping these patterns across competency domains, this review provides empirical support for calls to differentiate postlicensure IPE from student-focused models rather than assuming continuity across career stages.

From a methodological perspective, interpretation of these findings must be situated within the methodological characteristics and heterogeneity of the included studies. Although overall methodological quality was judged to be acceptable using JBI and Mixed Methods Appraisal Tool criteria, most studies used single-group pre-post or mixed methods designs, and no RCTs were identified. Outcome assessment relied predominantly on self-reported measures of attitudes, confidence, or perceived competence, with generally short follow-up periods. Such designs limit causal inference and are vulnerable to social desirability and recall biases, potentially overstating educational impact [44-46]. Moreover, substantial heterogeneity was observed across studies with respect to participant composition, clinical context, and IPE intensity, ranging from brief single-session workshops to multiweek interventions. While this heterogeneity reflects the diversity of real-world rehabilitation practice, it also constrains the comparability of outcomes and highlights the current lack of robust evidence evaluating sustained behavioral or organizational change in postlicensure IPE.

One of the most salient findings of this review is the relative paucity of IPE interventions explicitly targeting collaborative leadership, despite its recognition as a core competency in contemporary interprofessional frameworks [16,17]. Existing theories of workplace learning and continuing professional development emphasize that leadership and team coordination skills are most effectively cultivated through experiential, context-embedded learning rather than through didactic instruction alone [47,48]. However, our evidence gap mapping indicates that leadership-related competencies are rarely addressed using such approaches in rehabilitation-focused IPE. This misalignment may partly explain why improvements in attitudes or confidence do not consistently translate into sustained changes in clinical behavior [38,49]. By making this gap explicit, the present review contributes to the field by reframing leadership not as an ancillary outcome of communication training, but as a distinct educational target requiring intentional design and evaluation in postlicensure IPE.

Several limitations warrant consideration. The search strategy was restricted to 4 electronic databases and focused on English-language publications, which introduces a risk of publication bias and may limit the generalizability of the findings across different linguistic or cultural contexts. Due to technical constraints, certain comprehensive databases like Scopus were not included, potentially causing the review to miss relevant studies. Methodologically, the evidence base remains limited to a small number of studies, with a notable absence of RCTs, thereby constraining our ability to draw strong causal inferences. Most included studies relied heavily on self-reported outcomes and standardized questionnaires, which are susceptible to social desirability and recall biases. Such measures often capture “perceived” rather than “actual” change in competence and may not fully reflect objective changes in clinical behavior or team dynamics. Furthermore, the exclusion of standalone qualitative studies may have limited our depth of insight into the contextual influences and implementation processes that shape IPE in practice. Finally, as illustrated in our evidence gap map (Figure 1), the effectiveness of modalities such as simulation remains highly dependent on their alignment with specific interprofessional objectives rather than general instructional goals.

Future research should prioritize the development and evaluation of practice-integrated IPE models that move beyond short-term educational outcomes. Longitudinal and comparative study designs are needed to examine whether experiential learning strategies—such as team-based quality improvement initiatives, facilitated reflective practice, or shared leadership interventions—can produce sustained behavioral change among licensed rehabilitation professionals. Attention should be given to objective indicators of team functioning and decision-making in complex clinical contexts, rather than relying solely on self-reported measures. Additionally, future studies should explicitly account for professional mix, organizational context, and baseline team maturity, as these factors likely moderate IPE effectiveness. Addressing these gaps will be essential for generating more robust evidence base capable of informing institutional investment in IPE as a strategy for improving patient safety, workforce resilience, and the quality of rehabilitation care.

In conclusion, this scoping review adds a novel perspective by focusing exclusively on licensed rehabilitation professionals, distinguishing it from the vast body of existing literature centered on prelicensure students. It brings to the field a clearer understanding of the potential “leadership gap” and the current overreliance on didactic methods for experienced clinicians. The real-world implications suggest that, to move beyond short-term attitudinal change, professional-level IPE must transition toward systematic, practice-integrated models that address identified research gaps in leadership and team functioning. Real-world implementation should prioritize facilitator preparation and institutional support as critical components for sustainability. By fostering enduring improvements in health care team performance through longitudinal programs and objective behavioral measures, health care institutions can ultimately contribute to improvements in patient safety and the quality of rehabilitation care.

## Supplementary material

10.2196/76189 Multimedia Appendix 1 Search strategy.

10.2196/76189 Multimedia Appendix 2 Quality appraisal template.

10.2196/76189 Multimedia Appendix 3 Quality assessment of studies.

10.2196/76189 Multimedia Appendix 4 List of excluded studies.

10.2196/76189 Checklist 1 PRISMA 2020 checklist.

10.2196/76189 Checklist 2 PRISMA-ScR checklist.

10.2196/76189 Checklist 3 PRISMA-S checklist.

## Abbreviations

CINAHL: Cumulative Index to Nursing and Allied Health Literature
IPC: interprofessional collaboration
IPE: interprofessional education
JBI: Joanna Briggs Institute
OT: occupational therapist
PRISMA: Preferred Reporting Items for Systematic Reviews and Meta-Analyses
PRISMA-S: Preferred Reporting Items for Systematic Reviews and Meta-Analyses Search Reporting Extension
PRISMA-ScR: Preferred Reporting Items for Systematic Reviews and Meta-Analyses Extension for Scoping Reviews
PT: physical therapist
RCT: randomized controlled trial
SLP: speech-language pathologist
