# Supplementary material for: Educational Formats and Content Domains of Interprofessional Education for Licensed Rehabilitation Professionals: Scoping Review
Source: JMIR Med Educ. 2026 Mar 4;12:e76189. doi: 10.2196/76189 (PMC12978893; doi:10.2196/76189)
Supplement: Multimedia Appendix 1 [file mededu-v12-e76189-s001.docx]

**Multimedia Appendix 1. Search strategy**

PubMed

A comprehensive literature search was conducted using PubMed to identify studies related to interprofessional education and collaborative interventions involving rehabilitation professionals. The following Boolean search strategy was employed:

Search Terms:

• Interprofessional Concepts:

"interprofessional education" OR "interprofessional learning" OR "IPE" OR "IPL" OR

"interprofessional collaboration" OR "interprofessional work" OR "interprofessional practice" OR "interprofessional care" OR

"collaborative work" OR "collaborative practice" OR "collaborative care" OR

"team-based work" OR "team-based practice" OR "team-based care"

• Target Professions:

"occupational therap*" OR "physical therap*" OR "speech language therap*" OR "speech-language pathology" OR "rehabilitation" OR "OT" OR "PT" OR "ST"

• Intervention and Educational Components:

"intervention" OR "interventions" OR "interventive" OR

"methods" OR

"education" OR "training" OR "teaching" OR

"program" OR "programme"

• Outcomes of Interest:

"impact" OR "effect" OR "effectiveness" OR related terms

• Exclusion Criteria:

Studies primarily involving students, trainees, interns, residents, or faculty members were excluded using the following terms:

"student" OR "students" OR "learner" OR "trainee" OR "intern" OR "resident" OR "faculty"

Search Fields:

All terms were searched in [All Fields], with MeSH terms included where appropriate.

Search Logic:

The final search string was constructed using the Boolean operator:

(Interprofessional Concepts) AND (Target Professions) AND (Intervention/Education) AND (Impact/Effectiveness) NOT (Students/Trainees/Faculty)

Web of science

#1 (((ALL=("interprofessional education")) OR ALL=("interprofessional learning")) OR ALL=("IPE")) OR ALL=("IPL")

#2 (((ALL=("interprofessional collaboration")) OR ALL=("interprofessional work")) OR ALL=("interprofessional practice")) OR ALL=("interprofessional care")

#3 ((ALL=("collaborative work")) OR ALL=("collaborative practice")) OR ALL=("collaborative care")

#4 ((ALL=("team-based work")) OR ALL=("team-based practice")) OR ALL=("team-based care")

#5 (((((((ALL=("occupational therap*")) OR ALL=("physical therap*")) OR ALL=("speech-language therap*")) OR ALL=("speech-language pathology")) OR ALL=("rehabilitation")) OR ALL=("OT")) OR ALL=("PT")) OR ALL=("ST")

#6 (((ALL=(intervention)) OR ALL=(training)) OR ALL=(education)) OR ALL=(program)

#7 (ALL=(impact)) OR ALL=(effect)

#8 (((((ALL=(students)) OR ALL=(learners)) OR ALL=(trainees)) OR ALL=(interns)) OR ALL=(residents)) OR ALL=(faculty)

#9 #1 OR #2 OR #3 OR #4

#10 (#9 AND #6 AND #7) NOT #8

CINAHL and ERIC

S1 TX "interprofessional education" OR TX "interprofessional learning" OR TX "IPE" OR TX "IPL"

S2 TX "interprofessional collaboration" OR TX "interprofessional work" OR "interprofessional practice" OR "interprofessional care"

S3 TX "collaborative care" OR TX "collaborative practice" OR TX "collaborative work"

S4 TX "team-based care" OR TX "teams-based work" OR TX "team-based practice"

S5 TX "occupational therap*" OR TX "physical therap*" OR TX "speech-language therap*" OR TX "speech-language pathology" OR TX "rehabilitation" OR TX "OT" OR TX "PT" OR TX "ST"

S6 TX intervention OR TX training OR TX education OR TX program

S7 TX impact OR TX effect

S8 TX students OR TX learners OR TX trainees OR TX interns OR TX residents OR TX faculty

S9 S1 OR S2 OR S3 OR S4

S10 (S9 AND S5 AND S6 AND S7) NOT S8
