# Supplementary material for: Educational Formats and Content Domains of Interprofessional Education for Licensed Rehabilitation Professionals: Scoping Review
Source: JMIR Med Educ. 2026 Mar 4;12:e76189. doi: 10.2196/76189 (PMC12978893; doi:10.2196/76189)
Supplement: Multimedia Appendix 2 [file mededu-v12-e76189-s002.docx]

Multimedia Appendix 2. Quality appraisal template

JBI Checklist for Cross-Sectional Studies

Q1. Were the inclusion criteria clearly defined?

Q2. Were the study subjects and the setting described in detail?

Q3. Was the exposure measured in a valid and reliable way?

Q4. Were objective, standard criteria used for measurement of the condition?

Q5. Were confounding factors identified?

Q6. Were strategies to deal with confounding factors stated?

Q7. Were the outcomes measured in a valid and reliable way?

Q8. Was appropriate statistical analysis used?

JBI Checklist for Quasi-Experimental Studies

Q1. Is it clear what is the cause and what is the effect?

Q2. Were the participants included in any comparisons similar?

Q3. Were the participants included in any comparisons receiving similar treatment/care, other than the exposure or intervention of interest?

Q4. Was there a control group?

Q5. Were there multiple measurements of the outcome pre- and post-intervention/exposure?

Q6. Was follow-up complete, and if not, were differences between groups in terms of follow-up adequately described and analyzed?

Q7. Were the outcomes of participants included in any comparisons measured in the same way?

Q8. Was appropriate statistical analysis used?

MMAT for Mixed Methods Studies

Q1. Are there clear research questions?

Q2. Do the collected data allow addressing the research questions?

Q3. Is the mixed methods design relevant to address the research question?

Q4. Is the integration of qualitative and quantitative data/results relevant and adequately addressed?

Q5. Are the interpretations of the integrated results supported by the data?

Q6. Are divergences and inconsistencies between quantitative and qualitative results adequately addressed?

Q7. Do the different components of the study adhere to the quality criteria of each tradition of the methods involved?
