# Supplementary material for: Educational Formats and Content Domains of Interprofessional Education for Licensed Rehabilitation Professionals: Scoping Review
Source: JMIR Med Educ. 2026 Mar 4;12:e76189. doi: 10.2196/76189 (PMC12978893; doi:10.2196/76189)
Supplement: Multimedia Appendix 3 [file mededu-v12-e76189-s003.docx]

Multimedia Appendix 3. Quality assessment of studies

| ID | Author | Year | Appraisal Tool Used | Q1 | Q2 | Q3 | Q4 | Q5 | Q6 | Q7 | Q8 | Appraisal Summary |
| --- | --- | --- | --- | --- | --- | --- | --- | --- | --- | --- | --- | --- |
| 1 | Eskola et al. | 2024 | MMAT Mixed Methods | Yes | Yes | Yes | Yes | Yes | Yes | Yes | - | 7/7 Yes |
| 2 | Day et al. | 2022 | MMAT Mixed Methods | Yes | Yes | Yes | Yes | Yes | Yes | Yes | - | 7/7 Yes |
| 3 | Cohen et al. | 2021 | JBI Quasi-Experimental | Yes | N/A | Yes | No | Yes | Yes | Yes | Yes | 6/7 Yes |
| 4 | Zhang et al. | 2021 | JBI Quasi-Experimental | Yes | N/A | Yes | No | Yes | Yes | Yes | Yes | 6/7 Yes |
| 5 | Zhang et al. | 2021 | JBI Quasi-Experimental | Yes | Yes | Yes | Yes | Yes | Yes | Yes | Yes | 8/8 Yes |
| 6 | Zhang et al. | 2021 | JBI Quasi-Experimental | Yes | Yes | Yes | Yes | Yes | Yes | Yes | Yes | 8/8 Yes |
| 7 | Ober & Lape | 2019 | MMAT Mixed Methods | Yes | N/A | Yes | No | Yes | Yes | Yes | Yes | 6/7 Yes |
| 8 | Scuderi & Pain | 2019 | JBI Quasi-Experimental | Yes | N/A | Yes | No | Yes | Yes | Yes | Yes | 6/7 Yes |
| 9 | Sy | 2017 | JBI Cross-Sectional | Yes | Yes | Yes | Yes | No | No | Yes | Yes | 6/8 Yes |
| 10 | Phillips et al. | 2016 | JBI Quasi-Experimental | Yes | N/A | Yes | No | Yes | Yes | Yes | Yes | 6/7 Yes |
| 11 | Kenaszchuk et al. | 2011 | JBI Quasi-Experimental | Yes | N/A | Yes | No | Yes | Yes | Yes | Yes | 6/7 Yes |
