# Supplementary material for: Educational Formats and Content Domains of Interprofessional Education for Licensed Rehabilitation Professionals: Scoping Review
Source: JMIR Med Educ. 2026 Mar 4;12:e76189. doi: 10.2196/76189 (PMC12978893; doi:10.2196/76189)
Supplement: Multimedia Appendix 4 [file mededu-v12-e76189-s004.docx]

Multimedia Appendix 4. List of excluded studies

| Title | Author | Year | Reason for exclusion |
| --- | --- | --- | --- |
| Measuring the impact of interprofessional education on collaborative practice and patient outcomes | Cox, M | 2016 | Other |
| Improving chronic care through continuing education of interprofessional primary healthcare teams: a process evaluation | Paquette-Warren, J | 2014 | Wrong study design |
| A survey of pedagogical approaches and quality mechanisms used in education programs for mental health professionals | McCann, E | 2012 | Wrong study design |
| Broadening cultural sensitivity at the end of life: an interprofessional education program incorporating critical reflection | Halm, MA | 2012 | Wrong population |
| Implementation of unit-based interventions to improve teamwork and patient safety on a medical service | O'Leary, KJ | 2015 | Wrong population |
| Designing behavioral interventions using the capability-opportunity-motivation-behavior model and the theoretical domains framework to optimize oxygen saturation maintenance by NICU providers | Middleton, K | 2022 | Wrong population |
| Association of team-based primary care with health care utilization and costs among chronically ill patients | Meyers, DJ | 2019 | Wrong population |
| Impact of solution-focused coaching training on pediatric rehabilitation specialists: a longitudinal evaluation study. | Seko Y | 2020 | Wrong intervention |
| Learning together for effective collaboration in school-based occupational therapy practice. | Villeneuve MA | 2012 | Other |
| Building bridges in palliative rehabilitation: an evidence-based toolkit to promote collaboration. | Henshaw AM | 2024 | Other |
| Top ten tips palliative care clinicians should know about physical therapy, occupational therapy, and speech language pathology. | Habib MH | 2023 | Other |
| Implementing a collaborative coaching intervention for professionals providing care to children and their families: an exploratory study. | Tatla SK | 2017 | Wrong study design |
| Collaborative care transitions symposium: insights from participants. | Jeffs L | 2017 | Other |
| Educational outreach and collaborative care enhances physician's perceived knowledge about Developmental Coordination Disorder. | Gaines R | 2008 | Wrong population |
| Interprofessional mental health training in rural primary care: findings from a mixed methods study. | Heath O | 2015 | Wrong study design |
| Feasibility testing of an interprofessional education intervention to support collaborative practice in home care for older stroke survivors with multiple chronic conditions. | Bookey-Bassett S | 2024 | Other |
| Placing people in the same room is not enough: an interprofessional education intervention to improve collaborative knowledge of people with disabilities. | Ståhl C | 2016 | Wrong population |
| The evaluation of a multifaceted intervention to promote "speaking up" and strengthen interprofessional teamwork climate perceptions. | Ginsburg L | 2017 | Wrong population |
| Evaluation of a shared approach to interprofessional learning about stroke self-management. | Jones F | 2012 | Wrong population |
| Enhancing the value of integrated primary care: the role of occupational therapy. | Dahl-Popolizio S | 2016 | Wrong study design |
| Evaluating impact of a multi-dimensional education programme on perceived performance of primary care professionals in diabetes care. | Parekh S | 2015 | Wrong study design |
| Interprofessional continuing health education for diabetic patients in an urban underserved community. | Davis P | 2008 | Wrong study design |
| Re-examining the evaluation of interprofessional education for community mental health teams with a different lens: understanding presage, process and product factors. | Reeves S | 2006 | Wrong population |
| Towards healthy professional-client relationships: the value of an interprofessional training course. | Fronek P | 2009 | Wrong study design |
| Examining the effects of an obstetrics interprofessional programme on reductions to reportable events and their related costs. | Geary | 2018 | Wrong study design |
| Montreal Cross-Training Program: the contribution of positional clarification activities to help bridge fragmented prevention and treatment services for co-occurring disorders. | Perreault | 2020 | Wrong study design |
| Outcomes from an interprofessional curriculum on trauma-informed care among pediatric service providers. | Cerny | 2023 | Wrong population |
| The team leader coaching programme (TLCP) - a programme to implement team coaching in rehabilitation clinics - a feasibility study. | Küllenberg | 2021 | Wrong study design |
| A patient-centred team-coaching concept for medical rehabilitation. | Körner, M | 2018 | Wrong study design |
| Healthcare providers' perspectives on an interprofessional education intervention for promoting community re-engagement post stroke. | McKellar | 2011 | Wrong study design |
| How to know together? Physicians' co-orientation between hospitals and health centres. | Mertala S | 2009 | Wrong study design |
| IMPACT collaborative care improves depression in elderly patients in primary care in the longer term. | Gensichen J | 2006 | Other |
| Impact of interprofessional education on collaborative competencies of ABA and OT...Association of Schools Advancing Health Professions Annual Conference, October 19-21, 2022, The Westin Long Beach, Long Beach, California | Akselrud | 2023 | Other |
| Impact of interprofessional education on collaborative competencies of ABA and OT...The Association of Schools Advancing Health Professions, Annual Conference, October 17-19, 2023, Fort Lauderdale, Florida. | Akselrud | 2024 | Other |
| Improving interprofessional collaboration in a community setting: relationships with burnout, engagement and service quality. | Martinussen | 2012 | Wrong population |
| Improving teamwork climate in operating theatres: the shift from multiprofessionalism to interprofessionalism. | Bleakley A | 2006 | Wrong population |
| Influence of interactional structure on patient's participation during interprofessional discharge planning meetings in rehabilitation centers. | Schoeb | 2019 | Wrong study design |
| Using transprofessional care in the emergency department to reduce patient admissions: a retrospective audit of medical histories. | Morphet, Julia | 2016 | Wrong population |
| Prescription for education: development, evaluation, and implementation of a successful interprofessional education program for adults with inflammatory arthritis | Kennedy CA | 2011 | Wrong population |
| A pilot for understanding interdisciplinary teams in rehabilitation practice | White MJ | 2013 | Wrong study design |
| Improving teamwork, trust and safety: an ethnographic study of an interprofessional initiative | Jones, A | 2011 | Wrong population |
| Working together to deliver person-centred care within the stepped care model: an Australian multidisciplinary perspective. | Mareya, S | 2025 | Wrong study design |
| Re-examining interprofessional simulation: using social identity theory to explore the influence of 'profession' on interprofessional learning | Harrison, N | 2024 | Wrong study design |
| Changing the conversation: impact of guidelines designed to optimize interprofessional facilitation of simulation-based team training. | Ju M | 2024 | Wrong study design |
| Monitoring and evaluating an implementation strategy aimed at improving interprofessional collaboration in community-based fall prevention: a mixed-methods study. | van Scherpenseel MC | 2025 | Wrong study design |
| Facilitators and barriers to interprofessional collaboration among health professionals in primary healthcare centers in Qatar: a qualitative exploration using the "Gears" model. | El-Awaisi A | 2024 | Other |
